# Prognostic Risk Estimates of Patients with Multiple Sclerosis and Their Physicians: Comparison to an Online Analytical Risk Counseling Tool

**DOI:** 10.1371/journal.pone.0059042

**Published:** 2013-05-17

**Authors:** Christoph Heesen, Wolfgang Gaissmaier, Franziska Nguyen, Jan-Patrick Stellmann, Jürgen Kasper, Sascha Köpke, Christian Lederer, Anneke Neuhaus, Martin Daumer

**Affiliations:** 1 Institute for Neuroimmunology and Clinical MS Research and Department of Neurology, Hamburg, Germany; 2 Harding Center for Risk Literacy, Max Planck Institute for Human Development, Berlin, Germany; 3 Institute of Social Medicine, Nursing Research Group, University of Lübeck, Lübeck, Germany; 4 Sylvia Lawry Centre for Multiple Sclerosis Research & Human Motion Institute, Munich, Germany; Institute Biomedical Research August Pi Sunyer (IDIBAPS) - Hospital Clinic of Barcelona, Spain

## Abstract

**Background:**

Prognostic counseling in multiple sclerosis (MS) is difficult because of the high variability of disease progression. Simultaneously, patients and physicians are increasingly confronted with making treatment decisions at an early stage, which requires taking individual prognoses into account to strike a good balance between benefits and harms of treatments. It is therefore important to understand how patients and physicians estimate prognostic risk, and whether and how these estimates can be improved. An online analytical processing (OLAP) tool based on pooled data from placebo cohorts of clinical trials offers short-term prognostic estimates that can be used for individual risk counseling.

**Objective:**

The aim of this study was to clarify if personalized prognostic information as presented by the OLAP tool is considered useful and meaningful by patients. Furthermore, we used the OLAP tool to evaluate patients' and physicians' risk estimates. Within this evaluation process we assessed short-time prognostic risk estimates of patients with MS (final *n* = 110) and their physicians (n = 6) and compared them with the estimates of OLAP.

**Results:**

Patients rated the OLAP tool as understandable and acceptable, but to be only of moderate interest. It turned out that patients, physicians, and the OLAP tool ranked patients similarly regarding their risk of disease progression. Both patients' and physicians' estimates correlated most strongly with those disease covariates that the OLAP tool's estimates also correlated with most strongly. Exposure to the OLAP tool did not change patients' risk estimates.

**Conclusion:**

While the OLAP tool was rated understandable and acceptable, it was only of modest interest and did not change patients' prognostic estimates. The results suggest, however, that patients had some idea regarding their prognosis and which factors were most important in this regard. Future work with OLAP should assess long-term prognostic estimates and clarify its usefulness for patients and physicians facing treatment decisions.

## Introduction

Multiple sclerosis (MS) is a chronic neurological disease that can present in many different ways. In large cohort studies, overall prognosis shows an average walking distance of 100 m after 20 years of disease [1]. However, careful epidemiologic studies have indicated that up to 30% of MS courses might be benign [1], [2]. Despite many efforts to develop prognostic algorithms, making prognoses remains difficult, especially in the very early disease stage. Disease course is the only strong predictor, with primary progressive MS (PPMS) showing the worst course [3]. Relapse frequency and progression on the Expanded Disability Status Scale (EDSS) [4] are established measures of disease activity but their relevance for long-term prognosis is a matter of debate [5]–[7]. The large variability in disease progression is challenging, because patients and physicians are increasingly confronted with making treatment decisions at an early stage. Therefore, individual treatment decisions should ideally take individual prognoses into account to strike a good balance between benefits and harms of treatments.

The Sylvia Lawry Centre for Multiple Sclerosis Research (SLCMSR) has gathered data of placebo cohorts of randomized controlled trials (RCTs) between 1993 and 2003, observational studies, and natural history cohorts in the “Ian McDonald MS Database” comprising roughly 101,300 patient-years and data from approximately 26,700 patients. Recently, a Java-based online analytical processing (OLAP) tool has been developed by neurologists, information technology specialists, and biostatisticians using a matching algorithm to make individual risk estimates based on searches for matching patients from a subdatabase comprising placebo cohorts of RCTs [8].

Evidence-based patient information provides a framework for communicating medical evidence to patients [9]. Yet how trial data can be related to clinical care and individualized for a given patient for an individual treatment decision is a matter of discussion [10]. In several studies we have shown that open communication of scientific uncertainties is appreciated by MS patients [11]–[13]. But none of these existing information approaches is individualized.

Prognostic information is a sensitive issue and particularly at early stages of MS, anxiety and depression are highly relevant symptoms [14]. Wheelchair dependency in MS is an especially frightening risk for many people, which might even elicit avoidance behavior in perceiving this risk [15].

The aim of this study was to clarify if personalized prognostic information as presented by the OLAP tool offering short-term prognostic estimates is considered useful and meaningful by patients. Furthermore, we wanted to use the OLAP tool to evaluate patients' risk estimates and the estimates of their physicians. In particular, we investigated (i) whether the OLAP tool was understandable and acceptable to patients, (ii) to what extent patients' and physicians' estimates overall were in line with the estimates of the OLAP tool, (iii) whether patients' estimates changed after exposure to the OLAP tool, and (iv) whether patients', physicians', and the OLAP tool's estimates correlated similarly with various predictive disease covariates. These results show the applicability of the tool.

## Methods

Patients presenting at the MS outpatient unit of the Hamburg University Medical Center in 2009 were asked to participate if they had an EDSS score below 6. After informed consent was obtained each patient filled out a risk estimate questionnaire before and after presentation of the OLAP tool. In parallel, physicians were asked for their estimates concerning the same items without knowing patients' ratings and without information about the OLAP tool's estimates. Patients were led through the OLAP tool by a researcher (medical student experienced in interviewing MS patients). Patient and researcher together looked into the web-based tool filling in basic disease characteristics of the given patient. OLAP calculations were performed by the system and displayed on several graphics which were explained by the researcher. This process took about 30 minutes. MS demographic data were obtained on gender, age, age at diagnosis, EDSS, and ongoing immunotherapy. Physicians were neurologists in training (*n* = 5) and one senior neurologist (CH). This work is part of a larger work on informed shared decision making in MS that has been approved by the ethics committee of the chamber of physicians in Hamburg.

### Questionnaires

The questionnaire on risk perceptions was based on previous work [4], [15]. To estimate overall emotional threat, the risk of becoming wheelchair dependent was rated on a visual analog scale “0” meaning “not at all serious” and “100” meaning “the most serious thing which could happen”.

Patients were asked to give their estimates on the following disability milestones, which were also presented by the tool, and they were asked to indicate their estimates for a putative cohort of patients similar to themselves: (i) percentage of patients reaching a score of 6 or higher on the EDSS in the next year and in the following 3 years; and (ii) anticipated number of relapses in the next 6 and 18 months. We also compared patients' and physicians' estimates of other prognostic assumptions that were not included in the OLAP tool, such as the expected progression in the next year, and the proportion of 100 people like the patient who were estimated to become wheelchair dependent after 2 and 10 years, respectively.

After completing the OLAP tool, perception of the information was measured on four different dimensions with visual analogue scales (VAS): understanding, relevance, interest, and reassurance, as successfully applied in previous studies [12], [16]. The VAS scales were presented as 10 cm lines. Patients had to mark their evaluation with a pencil with a cross on the line. In the analysis each cm was transferred to a percentile. Therefore a rating at the 50% level of understanding means an intermediate position between complete understanding and no understanding at all.

Furthermore, we asked if patients had received prognostic counseling previously, if they would have liked more prognostic information earlier, and if they thought their clinical situation was adequately represented by the tool. Finally, we asked if they would have liked to have used the tool earlier and if they would recommend it to other patients.

### Database for Prognosis Calculation

One essential component of the OLAP tool is a matching algorithm. A given patient of interest is “identified” by a set of five disease covariates that are, at least in the short term, accepted as potential prognostic factors: disease course, current EDSS, age at diagnosis, disease duration, and the number of relapses in the last 12 months [1], [2], [17]. The matching algorithm then automatically selects the most similar subgroup of patients from the database, with similarity defined by the covariates on a disease course specific outcome. The tool displays the delineated possible EDSS evolution through the following 36 months in a graph. Other graphs show the expected proportion of similar patients who progress to an EDSS of 6 or higher across 36 months. All of these output data were used to compute for each patient who participated in the study the estimated percentage of similar patients with EDSS 6 or higher after 1 year and 3 years as well as the estimated relapses after 6 and 18 months, respectively. The tool gives annual relapse rates but graphically displays relapses after 6 and 18 months. We used this data to calculate the expected relapses within the next 6 months and within the next 18 months, respectively.

### Data Analysis Concept

Descriptive statistics were obtained on demographic data and perception of the information provided by the OLAP tool. Patients' and physicians' risk estimates were analyzed and compared to the estimates provided by the OLAP tool. The match between both patients' and physicians' estimates on the one hand and the OLAP tool's estimates on the other was assessed in two ways. First, we plotted estimates for patients (before and after exposure to the OLAP tool) and physicians as a function of the estimates provided by the OLAP tool. To more formally assess the match of the estimates, we followed Brown and Siegler's [18] suggestion and computed Spearman's rho rank correlation coefficients. To additionally assess how close those estimates were to actual OLAP tool estimates in absolute terms, we computed the absolute difference between estimates by patients (pre- and post-OLAP tool) and physicians and the estimates by the OLAP tool and compared those with Wilcoxon's signed-rank test. Nonparametric procedures in both cases were used, as the data were skewed.

Finally, we explored which disease covariates (i.e. demographic factors, disease course etc.) correlated with patients' and physicians' estimates. Here, we computed Spearman's rank correlation coefficient rho between prognostic covariates that also entered the OLAP tool matching algorithm and (i) patients' estimates before exposure to OLAP, (ii) patients' estimates after exposure to OLAP, (iii) physicians' estimates, and (iv) OLAP's estimates. These correlations reveal which disease covariates were most predictive of the rank order of risk estimates provided by the group of patients, the group of physicians, and the OLAP tool, respectively.

For the purpose of these analyses, course of the disease was given a value of 1 for clinically isolated syndrome (CIS) and relapsing remitting MS (RRMS) and a value of 2 for secondary progressive (SPMS) and primary progressive MS (PPMS). Relapse rates should be higher in the former category, while progression rates should be higher in the latter. We used Fisher's *z*-transformation for the calculation of confidence intervals of the correlation coefficients. We chose a non-parametric rank correlations between each single disease covariate and the estimates rather than including all of them at once into a multiple regression model for each of the groups, because many variables violated the distribution assumptions of multiple regression.

## Results

### Demographic Data

One hundred and fourteen MS patients agreed to participate. Four patients did not provide complete data and were excluded from subsequent analyses. Demographic data of the remaining 110 patients are displayed in table 1. Interestingly only seven patients (6%) reported that they received explicit prognostic counseling before participating in this study, and only 12 (11%) wanted more information about their putative prognosis.

**Table 1:** Demographic data of patients.

n	110
Female/male	80/30
CIS/RRMS/SPMS/PPMS	4/88/13/5
Mean age at onset of disease	31.7 (9.1)
Mean disease duration (years)	8.2 (7.0)
Mean EDSS	2.3 (1.4)
Relapses in preceding year 0/1/>1	63/33/14
On immunotherapy	46

Data are absolute numbers or mean values ± SD in brackets.

### Perception of the information provided by the OLAP Tool

**Figure 1:**
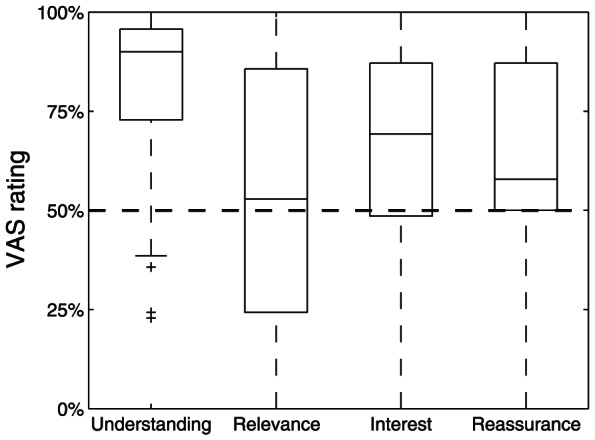
Perception of the information provided by the OLAP tool. Boxplots represent ratings on a VAS normalized to 0% to 100% with 50% representing a neutral rating. Extreme poles with pairs of adjectives are labeled to assess understanding (no understanding vs. complete understanding), relevance (not relevant vs. highly relevant), interest (not interesting vs. highly interesting), and threat (threatening vs. reassuring). Data as median and quartiles, outliers represented by plus signs.

In general, the OLAP tool was rated as highly acceptable (Figure 1). Understanding was also rated high, while patients did not perceive substantial threat. However, patients' interest was only modest, as was relevance. These results are also reflected in subsequent item responses (see table 2).

**Table 2:** Acceptance of the OLAP tool.

Item	yes	no	unsure
Agrees that OLAP matches clinical situation	53 (48%)	11 (10%)	43 (39%)
Would recommend OLAP to others	53 (48%)	20 (18%)	35 (32%)
Would have liked an earlier access to OLAP	17 (15%)	57 (52%)	34 (31%)

### Risk Estimates

#### Estimates of EDSS 6 or higher after 1 or 3 years

In general patients' estimates did not change substantially after exposure to the OLAP tool. Calculating absolute differences between patients' and physicians' estimates and the OLAP tool's estimates revealed that physicians' estimates were closer to the OLAP tool's than patients' estimates (Wilcoxon test p<0.001 and p = 0.008): patients' estimates differed by 12 (median: 5) percentage points (EDSS 6 in 1 year) and 18 (median: 10) percentage points (EDSS 6 in 3 years) from the OLAP tool's, physicians' estimates differed by 5 (median: 4) percentage points and 11 (median: 8) percentage points.

**Figure 2:**
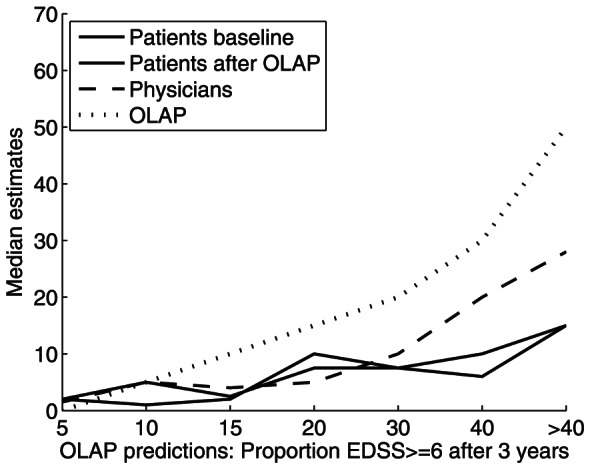
Estimates for EDSS ≥6 after 3 years. Mean estimates by patients at baseline and after exposure to the OLAP tool and by physicians, in relation to the OLAP tool's estimates are given. Median estimates for each of the groups can be read off the y-axis in relation to the predictions made by the OLAP tool (x-axis; binned into 7 categories, therefore not exactly bisecting the angle of x- and y-axis).

Estimates of patients and physicians were calibrated to the risk calculated by the OLAP tool, which is reflected in correlations between OLAP's estimates and estimates by patients (rho coefficients between 0.33 and 0.45) as well as physicians (rho coefficients between 0.59 and 0.62). Figure 2 depicts this calibration between OLAP estimates and estimates by patients (pre and post exposure to OLAP) and physicians for EDSS progression after 3 years. The figure clearly shows that both patients' and physicians' estimates increased as a function of OLAP estimates, but that physicians' estimates did so more strongly, and that patients showed severe underestimation.

#### Estimates of relapse frequency

Absolute differences to the OLAP tool's estimates were not different between physicians and patients for estimates of relapses within 6 month, but physicians were closer to OLAP for estimates of relapses within 18 months. Patients' estimates did not change through exposure to the OLAP tool. Absolute differences of patients' estimates to the OLAP tool were about half a relapse within 6 months (0.47 pre- and 0.45 post-OLAP tool exposure; medians: 0.45 and 0.45) and about one relapse (1.07 pre- and 1.07 post-OLAP tool exposure; medians_ 1.0 and 1.0) for 18 months. Physicians' estimates differed from the OLAP tool's as well by about half a relapse within 6-months (0.44; median: 0.45) and 0.70 (median: 0.65) relapses within 18-months. The calibration between OLAP estimates and estimates by patients (pre and post exposure to OLAP) and physicians for relapses after 18 months was slightly worse than calibration regarding OLAP tool estimates of EDSS 6 or higher in terms of rank correlations (rho = 0.26–.41 vs. rho = 0.33–.62).

#### Estimates of other progression measures

The mean perceived threat of wheelchair dependency was 71 out of 100 meaning wheelchair dependency as “the most serious thing which could happen”. Among patients, the OLAP tool only changed one out of three estimates (table 3): The estimated proportion of 100 patients who will be wheelchair dependent within 10 years was slightly lower after OLAP tool exposure than before, even though the median was the same (5% in both cases; means:11% and 14.6%), indicating that OLAP particularly reduced some outlier estimates. Physicians' estimates of percentage of wheelchair dependency after 10 years were substantially higher (median: 10%; mean:18%).

**Table 3:** Patients' and physicians' estimates of other progression measures.

	Patients pre–OLAP n = 110	Patients post–OLAP n = 110	p* pre–OLAP vs. post–OLAP	Physicians n = 92	p* Patients pre–OLAP vs. Physicians	p*Patients post–OLAP vs. Physicians
Progressing next year (Likert 0–4)	0 (0–1)	0 (0–1)	0.147	1 (0–1)	0.557	0.102
% wheelchair dependent in 2 years	1 (0–5)	1 (0–5)	0.798	1 (0–7.25)	0.382	0.444
% wheelchair dependent in 10 years	5 (0–16.25)	5 (0.75–15)	0.002	10 (4.25–22.75)	0.036	0.001

Data represent medians with the interquartile range in brackets.

* P-values based on Wilcoxon signed-rank tests.

#### Correlations between estimates and disease covariates

**Figure 3:**
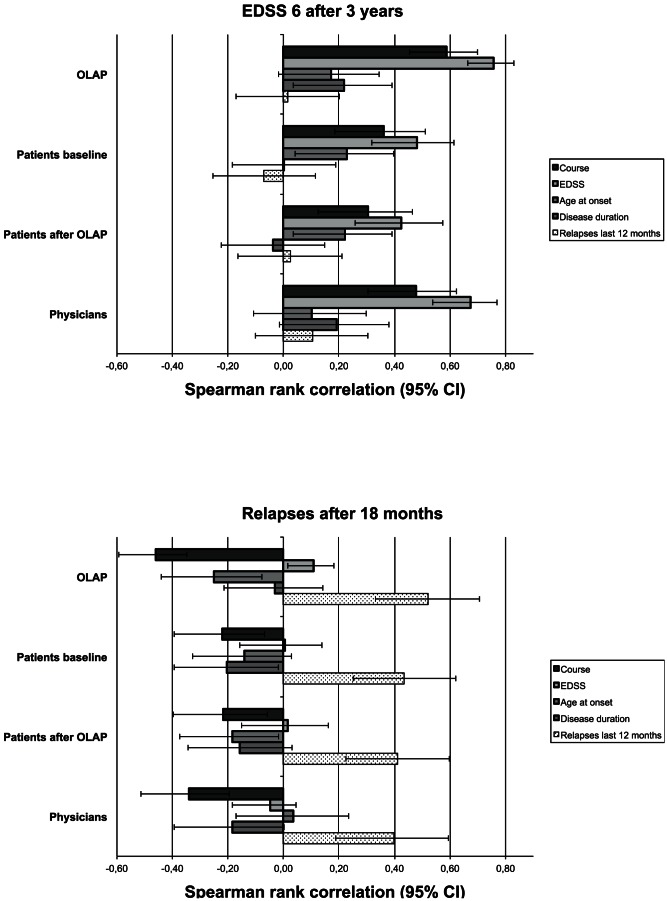
Correlations between risk estimates and disease covariates. Data are shown separately for patients (at baseline and after exposure to the OLAP tool), physicians, and the OLAP tool. Patients' and physicians' estimates correlated most strongly with those factors that also correlated most strongly with the OLAP tool's predictions, both regarding the risk of EDSS 6 or higher after 3 years (upper panel) and relapses after 18 months (lower panel). Note: Course of the disease was given a value of 1 for CIS and RRMS and a value of 2 for SPMS and PPMS.

The OLAP tool's estimates and patients' as well as physicians' estimates show a strong agreement with regard to the covariates they correlate with (Figure 3). In the case of risk estimates for EDSS 6 or higher after 3 years (Figure 3, upper panel), the two most important factors were the current EDSS score and the course of the disease. Regarding disease course, the positive correlation shows that the estimates were higher for SPMS and PPMS than for CIS and RRMS. There was no difference with regard to patients before or after exposure to the OLAP tool. Physicians' estimates were more similar to those of the OLAP tool as they correlated more strongly with disease course and EDSS than those of patients.

In the case of risk estimates for relapses within the next 18 months (Figure 3, lower panel), the two most important factors were relapses within the last 12 months and disease course. Regarding the latter, the negative correlation shows that the estimates were higher for CIS and RRMS than for SPMS and PPMS. Both patients' and physicians' estimates correlated similarly with those two factors. Again, there was no difference with regard to patients before or after exposure to the OLAP tool.

## Discussion

This study is the first comparing prognostic estimates from MS patients and their physicians with an online analytical processing (OLAP) tool. We studied a heterogeneous but only mildly disabled group of consecutive MS patients of a university-based outpatient clinic.

As a major result, patients, physicians, and the OLAP tool ranked patients similarly regarding the risk of having an EDSS of 6 or higher after 1 year and 3 years, respectively. The calibration with the OLAP tool in terms of rank correlations did not differ between 1-year estimates and 3-year estimates but was better for physicians than for patients. Patients' and physicians' estimates of relapses in the following 6 and 18 months were also calibrated with the OLAP tool's estimates, although somewhat lower in terms of rank correlations. Exposure to the OLAP tool did not substantially change any of the patients' estimates for which an OLAP tool estimate was available.

Both patients' and physicians' estimates correlated most strongly with those prognostic covariates that the OLAP tool's estimates also correlated with most strongly. These were disease course and baseline EDSS for progression rates, which is in line with the current epidemiological literature [1], [19]. The most important relapse rate predictors were relapses within the last 12 months and disease course. These factors have also been found relevant in analyses of placebo cohorts in MS randomized controlled trials [20], [21]. Our findings suggest that patients and physicians were able to pick up the most important prognostic information, and physicians somewhat more so than patients.

In summary, patients and physicians are similar in their short-term (up to 3 years) risk estimates. Patients' estimates correlated with the same disease covariates as did their physicians' and the OLAP tool's estimates, which suggests that patients had at least some idea regarding which factors were most important for their prognosis if one refers to the general evidence on prognostic factors [1]. However, even though the tool was rated highly understandable and not threatening, relevance and interest were only rated modest among patients. Only 11% said that they would like more information beyond what the OLAP tool could provide about their possible prognosis, and only 48% would recommend the tool for other patients. Finally, the OLAP tool did not substantially change patients' estimates.

This ambivalence toward the tested version of the OLAP tool might be because it only provides short-term prognostic estimates. Future extensions of the OLAP tool will implement long-term natural history as well as treatment data if available. As just recently 16-year treatment data of the first interferon-β trial have become available [22], estimates of long-term treatment effects might also be available for matching algorithms. Adding paraclinical measures such as, for instance, magnetic resonance imaging in further OLAP developments might help to further improve prognostic estimates for patient counseling.

In general, prognostic information is highly demanded when surveying patients' needs [11], and our study indicates that a large number of patients (here more than two-thirds) had not received explicit prognostic counseling previously. In this regard, patients' disease stages may be relevant, as especially decisions about powerful drugs that come along with severe side effects require serious progression estimates. However, in clinical practice the demand for prognostic information seems uncertain, as indicated by our data on acceptability and interest. Compared to results of previous studies, the demand for further information and the proportion who recommended the tool to other patients were markedly lower for the OLAP tool [11], [16], [23].

Therefore, we conclude with the question of whether patients really want to know their most realistic prognosis as precisely as possible and, if so, how this prognosis should best be communicated to them. The OLAP tool approach for short-term prognostic counseling that we studied here has raised no safety concerns and indicates the overall acceptance of the approach by patients. Future studies of OLAP tools will use long-term prognostic information to answer the question about the prognostic accuracy of the tool and physicians' estimates, which was beyond the scope of the current study. In addition different ways of presenting individualized prognostic information to patients need to be tested.

## References

[1] Degenhardt A, Ramagopalan SV, Scalfari A, Ebers GC (2009) Clinical prognostic factors in multiple sclerosis: a natural history review. Nature Reviews Neurology 5: 672–682. 19953117 10.1038/nrneurol.2009.178

[2] Pittock SJ, McClelland RL, Mayr WT, Jorgensen NW, Weinshenker BG, et al (2004) Clinical implications of benign multiple sclerosis: a 20-year population-based follow-up study. Annal Neurol 56: 303–306. 15293286 10.1002/ana.20197

[3] Vukusic S, Confavreux C (2007) Natural history of multiple sclerosis: risk factors and prognostic indicators. Cur Op Neuro 20: 269–274. 10.1097/WCO.0b013e32812583ad 17495619

[4] Kurtzke JF (1983) Rating neurologic impairment in multiple sclerosis: an expanded disability status scale (EDSS). Neurology 33: 1444–52. 6685237 10.1212/wnl.33.11.1444

[5] Scalfari A, Neuhaus A, Degenhardt A, Rice GP, Muraro PA, et al (2010) The natural history of multiple sclerosis: a geographically based study 10: relapses and long-term disability. Brain 133: 1914–1929. 20534650 10.1093/brain/awq118 PMC2892939

[6] Tremlett H, Zhao Y, Rieckmann P, Hutchinson M (2010) New perspectives in the natural history of multiple sclerosis. Neurology 74: 2004–2015. 20548045 10.1212/WNL.0b013e3181e3973f

[7] Young PJ, Lederer C, Eder K, Daumer M, Neiss A, et al (2006) Relapses and subsequent worsening of disability in relapsing-remitting multiple sclerosis. Neurology 6: 804–808. 10.1212/01.wnl.0000234064.17156.03 16966541

[8] Daumer M, Neuhaus A, Lederer C, Scholz M, Wolinsky JS, et al (2007) Prognosis of the individual course of disease—steps in developing a decision support tool for multiple sclerosis. BMC Medical Informatics and Decision Making 7: 11–16. 17488517 10.1186/1472-6947-7-11 PMC1884137

[9] Bunge M, Mühlhauser I, Steckelberg A (2010) What constitutes evidence-based patient information? Overview of discussed criteria. Pat Ed Counsel 78: 316–328. 10.1016/j.pec.2009.10.029 20005067

[10] Rothwell PM (2005) Treating individuals. External validity of randomised controlled trials: “To whom do the results of this trial apply?”. Lancet 36: 82–93. 10.1016/S0140-6736(04)17670-8 15639683

[11] Heesen C, Kasper J, Segal J, Koepke S, Mühlhauser I (2004) Decisional role preferences, knowledge and information interests in patients with multiple sclerosis. Mult Scl 10: 643–650. 10.1191/1352458504ms1112oa 15584489

[12] Kasper J, Köpke S, Mühlhauser I, Heesen C (2006) Evidence-based patient information about treatment of multiple sclerosis—a phase one study on comprehension and emotional responses. Pat Ed Counsel 62: 56–63. 10.1016/j.pec.2005.06.002 16098706

[13] Heesen C, Schäffler N, Kasper J, Mühlhauser I, Köpke S (2009) Suspected multiple sclerosis—what to do? Evaluation of a patient information leaflet. Mult Scler 15: 1103–1112. 19625332 10.1177/1352458509106508

[14] Janssens AC, van Doorn PA, de Boer JB, van der Mech FG, Passchier J (2004) Perception of prognostic risk in patients with multiple sclerosis: the relationship with anxiety, depression, and disease-related distress. J Clin Epidem 57: 180–186. 10.1016/S0895-4356(03)00260-9 15125628

[15] Boeije HR, Janssens CJW (2004) ‘It might happen or it might not’: how patients with multiple sclerosis explain their perception of prognostic risk. Soc Sci Med 59: 861–868. 15177841 10.1016/j.socscimed.2003.11.040

[16] Heesen C, Kleiter I, Nguyen F, Schäffler N, Kasper J, et al (2010) Risk perception in natalizumab-treated multiple sclerosis patients and their neurologists. Mult Scler 16: 1507–1512. 20826527 10.1177/1352458510379819

[17] Confavreux C, Vukusic S, Moreau T, Adeleine P (2000) Relapses and progression of disability in multiple sclerosis. N Engl J Med 343: 1430–1438. 11078767 10.1056/NEJM200011163432001

[18] Brown NR, Siegler RS (1993) Metrics and mappings: a framework for understanding real-world quantitative estimation. Psycholl Rev 100: 511–534. 10.1037/0033-295x.100.3.511 8356188

[19] Tremlett H, Zhao Y, Rieckmann P, Hutchinson M (2010) New perspectives in the natural history of multiple sclerosis. Neurology 74 24: 2004–15. 20548045 10.1212/WNL.0b013e3181e3973f

[20] Inusah S, Sormani MP, Cofield SS, Aban IB, Musani SK, et al (2010) Assessing changes in relapse rates in multiple sclerosis. Mult Scler 16: 1414–1421. 20810517 10.1177/1352458510379246

[21] Stellmann JP, Herich L, Pöttgen J, Schippling S, Martin R (2010) Relapses and EDSS—progression of placebo cohorts in MS phase III trials in the last 20 years. Mult Scler 16 10 Suppl: S210.

[22] Ebers GC, Traboulsee A, Li D, Langdon D, Reder AT, et al (2010) Analysis of clinical outcomes according to original treatment groups 16 years after the pivotal IFNB-1b trial. Journal of Neurology, Neurosurgery & Psychiatry 81: 907–912. 10.1136/jnnp.2009.204123 20562430

[23] Heesen C, Segal J, Reich C, Hämäläinen P, Broemel F, et al (2006) Patient information on cognitive symptoms in multiple sclerosis—acceptability in relation to disease duration. Acta Neurol Scand 11: 268–272. 10.1111/j.1600-0404.2006.00630.x 16942547

